# Driving the expression of the *Salmonella enterica* sv Typhimurium flagellum using *flhDC* from *Escherichia coli* results in key regulatory and cellular differences

**DOI:** 10.1038/s41598-018-35005-2

**Published:** 2018-11-12

**Authors:** Ayman Albanna, Martin Sim, Paul A. Hoskisson, Colin Gillespie, Christopher V. Rao, Phillip D. Aldridge

**Affiliations:** 1 Centre for Bacterial Cell Biology, Baddiley Clark Building, Newcastle University, Richardson Road, Newcastle upon Tyne, NE2 4AX UK; 2 Institute for Cell and Molecular Biosciences, Newcastle University, Framlington Place, Newcastle upon Tyne, NE2 4HH UK; 3 Strathclyde Institute of Pharmacy and Biomedical Sciences, University of Strathclyde, Glasgow, G4 0RE UK; 4 School of Mathematics & Statistics, Herschel Building, Newcastle University, Newcastle upon Tyne, NE1 7RU UK; 5 Department of Chemical and Biomolecular Engineering, University of Illinois at Urbana-Champaign, Urbana, Illinois 61801 United States; 6 College of Environmental Science & Technology, Mosul University, Mosul, 41002 Iraq; 7 Present Address: Isomerase Therapeutics Ltd., Chesterford Research Park, Cambridge, CB10 1XL UK

## Abstract

The flagellar systems of *Escherichia coli* and *Salmonella enterica* exhibit a significant level of genetic and functional synteny. Both systems are controlled by the flagellar specific master regulator FlhD_4_C_2_. Since the early days of genetic analyses of flagellar systems it has been known that *E. coli flhDC* can complement a ∆*flhDC* mutant in *S. enterica*. The genomic revolution has identified how genetic changes to transcription factors and/or DNA binding sites can impact the phenotypic outcome across related species. We were therefore interested in asking: using modern tools to interrogate flagellar gene expression and assembly, what would the impact be of replacing the *flhDC* coding sequences in *S. enterica* for the *E. coli* genes at the *flhDC S. entercia* chromosomal locus? We show that even though all strains created are motile, flagellar gene expression is measurably lower when *flhDC*_EC_ are present. These changes can be attributed to the impact of FlhD_4_C_2_ DNA recognition and the protein-protein interactions required to generate a stable FlhD_4_C_2_ complex. Furthermore, our data suggests that in *E. coli* the internal flagellar FliT regulatory feedback loop has a marked difference with respect to output of the flagellar systems. We argue due diligence is required in making assumptions based on heterologous expression of regulators and that even systems showing significant synteny may not behave in exactly the same manner.

## Introduction

The flagellum in the enteric bacteria, *Escherichia coli* and *Salmonella enterica*, has been studied extensively for over fifty years and provides the canonical example for bacterial motility. These studies have revealed not only the complex structure of the enteric flagellum but also its role in host colonization, pathogenesis, and cellular physiology^1–4^. In addition, these studies have identified many of the complex regulatory processes that coordinate the assembly and control of this exquisitely complex biological machine^3–5^.

The flagellum in *E. coli* and *S. enterica* are structurally very similar and are often tacitly assumed to be effectively identical aside from differences in the filament structure. However, in the case of regulation, these assumptions are based more on sequence similarity rather than on actual experimental data^5,6^. Indeed, a number of studies have shown that these two systems are regulated in entirely different manners in response to environmental signals despite strong gene synteny. For example, many common *E. coli* strains are motile only during growth in nutrient-poor conditions whereas many common *S. enterica* strains are motile only during growth in nutrient-rich conditions^7^. In addition, *E. coli* is more motile at 30 °C than at 37 °C whereas motility *S. enterica* is generally insensitive to these temperature differences^8^. *E. coli flhDC* are transcribed from a single transcriptional start site that is responsive to OmpR, RcsB and CRP regulation, to name only a few regulatory inputs^8^. In contrast *S. enterica flhDC* transcription is significantly more complex with up to 5 transcriptional start sites, albeit with only a subset being responsible for the majority of *flhDC* transcription^9^.

Part of the problem is that different questions have been asked when studying the regulation of motility in these two bacterial species. Most studies in *E. coli* have focused on the environmental signals and associated regulatory process that induce bacterial motility. In particular, they have focused on the processes that regulate the expression of the master flagellar regulator, FlhD_4_C_2_^8^. Most studies in *S. enterica*, on the other hand, have focused on the regulatory processes that coordinate the assembly process following induction^4^. In particular, they have focused on the downstream regulatory processes induced by FlhD_4_C_2_^3^.

Despite differences in regulation, the protein subunits of master flagellar regulators, FlhC and FlhD, exhibit high sequence similarity sharing 94 and 92% identity, respectively (Figure S1), between *E. coli* and *S. enterica*. For both proteins the most significant amino acid changes are within the last 8 amino acids. Other substitutions are scattered across each protein and do not provide a consistent mutational pattern that provide a clear phenotypic explanation. Given that modifications to transcription factors and/or promoter structure can lead to divergence in regulatory circuits^10^, we were interested in how FlhD_4_C_2_ functions in different genetic backgrounds. Previously, it was shown that *E. coli flhDC* can complement a ∆*flhDC* mutant in *S. enterica*, suggesting that these proteins are functionally identical in the two bacterial species^11^. However, it is not clear whether they are regulated in the same manner. We, therefore, investigated the impact of replacing the native master regulator in *S. enterica* with the one from *E. coli*. Defining the impact of known FlhD_4_C_2_ regulators such as ClpP, RflP (previously known as YdiV), FliT and FliZ on the two complexes suggest that these two species have adapted in how they perceive FlhD_4_C_2_. We argue that these phenotypic differences arise from adaptations *E. coli* and *S. enterica* have made during evolution to expand or modify cellular function with respect to movement within specific environmental niches.

## Results

### Orthologous flhDC from *E. coli* can functionally complement flhDC in *S. enterica*

Given the similarities between the flagellar systems in *S. enterica* and *E. coli*, we sought to determine whether the FlhD_4_C_2_ master regulator is functionally equivalent in these two species of bacteria. To test this hypothesis, we replaced the *flhDC* genes in *S. enterica* (*flhDC*_SE_) with the *flhDC* genes from *E. coli* (*flhDC*_EC_). The reason that we performed these experiments in *S. enterica* rather than *E. coli* was that the flagellar system is better characterized in the former, particularly with regards to transcriptional regulation. To avoid plasmid associated artefacts associated with the ectopic expression of *flhDC*, we replaced the entire *S. enterica flhDC* operon with the *flhDC* operon from *E. coli* at the native chromosomal locus (Figure S2).

**Figure 1:**
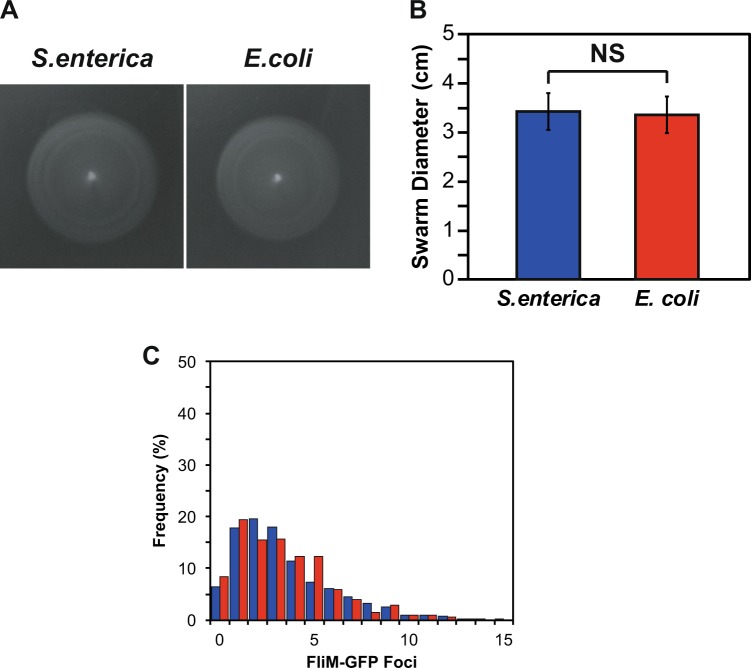
(**A**) Motility of *flhDC*_*ST*_ and *flhDC*_*EC*_ driven by P_*flhDC*_. (**B**) Quantification of swarms produced in motility agar after 6 to 8 hours incubation. Error bars indicate calculated standard deviations. (**C**) Percentage frequency of FliM-GFP foci for *flhDC*_EC_ compared to *S. enterica* with *flhDC* under the control of P_*flhDC*_. Colors of bars in the graph correspond to the source of *flhDC* as shown in (**B**).

We first tested whether *flhDC*_EC_ was motile as determined using soft-agar motility plates. As shown in Fig. 1A and B, these strains formed rings similar to the wild type. These results demonstrate that *flhDC*_EC_ is functional in *S. enterica*. However, motility plates measure both motility and chemotaxis and do not provide any insights regarding possibly changes in the number of flagella per cell. To determine the impact *flhDC*_EC_ had upon flagellar numbers we used a FliM-GFP fusion as a proxy for flagellar numbers (Fig. 1C). When this fluorescent protein fusion is expressed in cells, it forms spots associated with nascent C-rings that loosely correlate with the number of flagella^12–14^. By counting the number of spots per cell, we can determine the number of flagella made per cell. As shown in Fig. 1C, *flhDC*_EC_ did not change flagellar numbers as compared to the wild type. These results demonstrate *flhDC*_EC_ induces flagellar gene expression at similar levels as the wild type.

### flhDC requires a specific transcription rate to maintain optimal flagellar numbers

**Figure 2:**
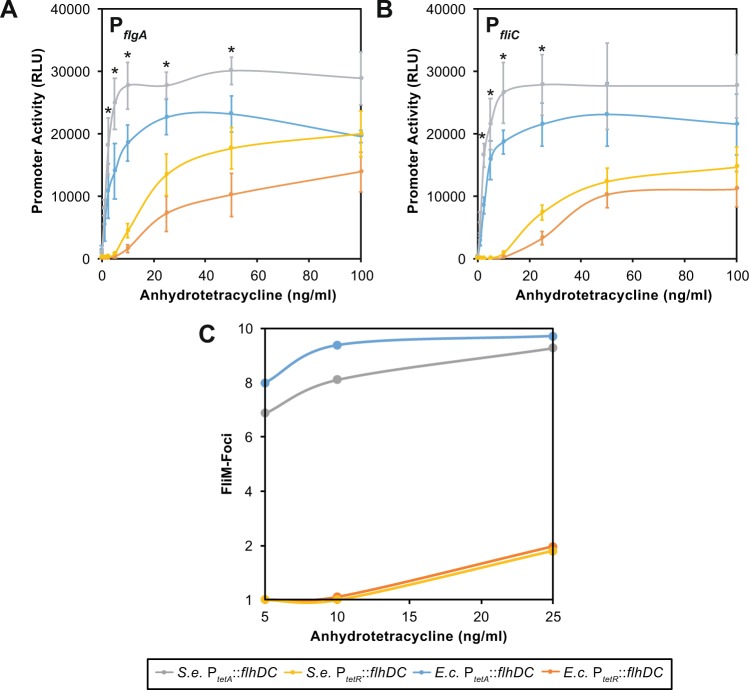
Titration of P_*tetA*_*::flhDC*_*ST/EC*_ and P_*tetR*_*::flhDC*_*ST/EC*_ activity suggests a given rate of transcription drives optimal flagellar assembly. (**A**) Activity of P_*flgA*_ in response to P_*tetA*_ or P_*tetR*_ transcription of *flhDC* from *S. enterica* (*S.e*.) or *E. coli* (*E.c*.). Data sets that exhibit statistical significance at P < 0.03 are shown with ‘*’. Using 10 ng anhydrotetracycline as an example, due to this being where FlhD_4_C_2SE_ reaches maximal activity via P_*tetA*_ expression, the following comparisons are significant: *S.e*. P_*tetA*_ v P_*tetR*_ (P = 0.008) *E.c*. P_*tetA*_ v P_*tetR*_ (P = 0.009), while *S.e*. P_*tetA*_ v *E.c*. P_*tetA*_ is not (P = 0.186). Error bars show the standard error of the mean. (**B**) Activity of P_*fliC*_ in response to P_*tetA*_ or P_*tetR*_ transcription of *flhDC*. As in (A) the ‘*’ identifies data sets that exhibit ANOVA statistical significance at P < 0.005. In agreement with P_*flgA*_ activity P_*tetA*_ v P_*tetR*_ ANOVA comparisions were significant for 5 to 25 ng anhydrotetracycline (e.g. at 10 ng *S.e*.: P = 0.012; *E.c*.: P = 0.002) while *S.e*. P_*tetA*_ v *E.c*. P_*tetA*_ comparisons were not. Error bars show the standard error of the mean. **C**. flagellar numbers as defined by FliM-foci in response to P_*tetA*_ or P_*tetR*_ transcription of *flhDC*. In agreement with the statistical analysis FliM-Foci profiles reflect the statistical significance associated with the expression data shown in (**A**) and (**B**). All data represents the analysis of gene expression or FliM-Foci from 3 independent repeats. FliM-Foci data is based on n > 400 cells for each data point. The colours of lines reflect the strains used in Figs 4, 5 and 6, for example in these figures, when graphs are used, *S.e. flhDC* is represented as gray and its *E.c. flhDC* replacement as light blue.

The flagellar network in *S. enterica* contains a number of feedback loops to ensure that the cells regulate the number of flagella produced^4^. One possibility is that these feedback loops mask any differences in FlhD_4_C_2EC_ activity. To test this hypothesis, we replaced the native P_*flhD*_ promoter with the tetracycline-inducible P_*tetA*_/_*tetR*_ promoters. We then measured flagellar gene expression using a luciferase reporter system^15^. In this case, a consistent and significant change (e.g at 10 ng for P_*flgA*_ ANOVA P = 0.0008) in flagellar gene expression was observed when comparing activity across all strains tested (Fig. 2A and B). Maximal expression of P_*flgA*_ and P_*fliC*_, chosen to reflect flagellar gene expression at different stages of flagellar assembly^5^, for both complexes was observed between 10 and 25 ng/ml of anhydrotetracycline, when *flhDC* transcription was from P_*tetA*_ (Fig. 2A and B). In contrast, P_*tetR*_, the weaker of the two tetracycline inducible promoters, reached a maximal output between 50 to 100 ng/ml anhydrotetracycline. When comparing P_*tetA*_ and P_*tetR*_ activity around the transition points in each experiment, for example 10 ng anhydrotetracycline for P_*flgA*_, the difference between P_*tetA*_ and P_*tetR*_ expression was significant (see Fig. 2 legend for P-values). However, the observed differences between FlhD_4_C_2EC_ to FlhD_4_C_2SE_ for either P_*tetA*_ or P_*tetR*_ expression were not significant (e.g. at 10 ng for P_*flgA*_ via P_*tetA*_ expression ANOVA P = 0.186).

We also measured the number of FliM-GFP foci at different anhydrotetracycline concentrations. P_*tetR*_::*flhDC* expression generated on average of approximately two FliM-foci per cell at 25 ng/ml of anhydrotetracycline for both FlhD_4_C_2_ complexes (Fig. 2C). In contrast, 5 ng/ml induction of the P_*tetA*_::*flhDC*_*EC*_ strain was sufficient to generate typical FliM-foci numbers (approx. 8 flagellar foci per cell). These data reflect the statistical significance of the expression data where a marked difference between P_*tetA*_ and P_*tetR*_ expression was observed (Fig. 2A and B). Even with the strong decrease in average foci per cell at these levels of induction for P_*tetR*_, the number of basal bodies observed is sufficient to allow motility at comparable levels in the motility agar assay (Figure S3).

### Replacement of flhC but not flhD in S. enterica with the *E. coli* orthologs affects motility

**Figure 3:**
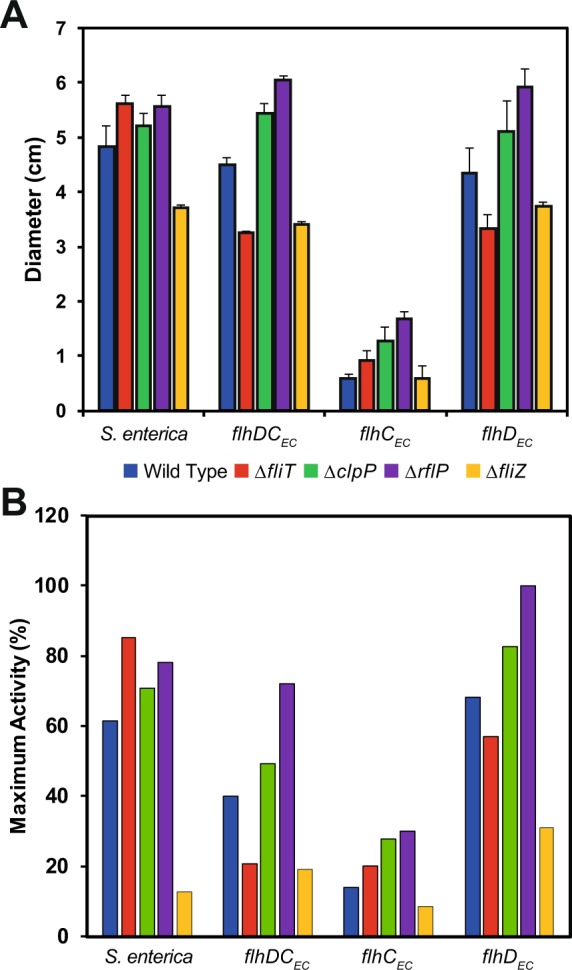
Motility phenotypes and gene expression of *flhDC*_ST_, *flhDC*_EC_, *flhD*_EC_ and *flhC*_EC_ strains in the absence of known FlhD_4_C_2_ regulators. (**A**) Quantification of n = 3 swarms per strain produced in motility agar after 6 to 8 hours incubation at 37 °C. Error bars indicate calculated standard deviations. (**B**) Relative activity of P_*fliC*_ in all strains as a percent of the maximal activity observed in *flhD*_EC_ ∆*rflP*.

The hetero-oligomeric regulator FlhD_4_C_2_ is unusual in bacteria as the majority of transcriptional regulators are believed to be homo-oligomeric complexes. To determine the relative contributions of the two subunits, we individually replaced the *flhC* or *flhD* genes from *S. enterica* with their ortholog from *E. coli* (Figure S2). When we tested the two strains using motility plates, we found that motility was inhibited in the strain where *flhC*_EC_ replaced the native *S. enterica flhC* (Fig. 3A; blue bars), with an 88% reduction in swarm diameter when compared to WT *S. enterica*. The introduction of *flhD*_EC_ compared to *flhDC*_EC_ or *flhDC*_SE_ produced swarms of a comparable size (Fig. 3A; blue bars).

**Figure 4:**
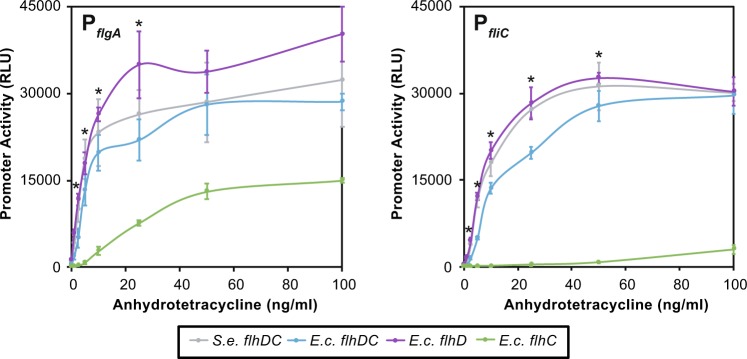
Titration of P_*tetA*_*::flhDC* for *S. enterica*, *flhDC*_EC_, *flhD*_EC_ and *flhC*_EC_ suggests that *flhC*_EC_ exhibits low motility due reduced P_*flgA*_ activity and a strong reduction in P_*fliC*_ activity. Note that the legend indicates which gene has been replaced compared to *S.e. flhDC*. The colours of lines reflect the strains used in Figs 2, 5 and 6, for example in all figures *S.e. flhDC* is represented as gray and the *E.c. flhDC* replacement as light blue. Inducible expression was driven from the P_*tetA*_ promoter within the TetRA cassette of Tn10 as in Fig. 2. Data represents n = 3 independent repeats of the expression assays. Data sets exhibiting ANOVA statistical significance of P < 0.03 are indicated with a ‘*’. Error bars show the standard error of the mean. The P_*flgA*_ variation observed between *S.e. flhDC*, *E.c. flhDC* and *E.c. flhD* at 10 and 25 ng was not significant (ANOVA P = 0.64 and 0.33 respectively). In agreement for P_*fliC*_ data *S.e. flhDC*, *E.c. flhDC* and *E.c. flhD* exhibits ANOVA P-values of 10 ng: 0.16 and 25 ng: 0.07. All ANOVA statistical comparisons to *E.c. flhC* were significant P < 0.04.

Using the dose-dependent inducible P_*tetA*_ promoter^16^ we observed that P_*tetA*_ expression of *flhC*_EC_ led to reduced P_*flgA*_ transcription and strongly reduced P_*fliC*_ transcription (Fig. 4). Strains expressing *flhD*_EC_ in *S. enterica* showed a mild increase in P_*flgA*_ gene expression and a similar response for P_*fliC*_, although these changes were not significant (see Fig. 4 for P values). These data suggest that the combination of FlhD_SE_ and FlhC_EC_ generates an inefficient FlhD_4_C_2_ complex, resulting in reduced motility.

### Orthologous FlhC and FlhD interaction is species specific and a key determinant of promoter recognition by the FlhD_4_C_2_ complex

**Figure 5:**
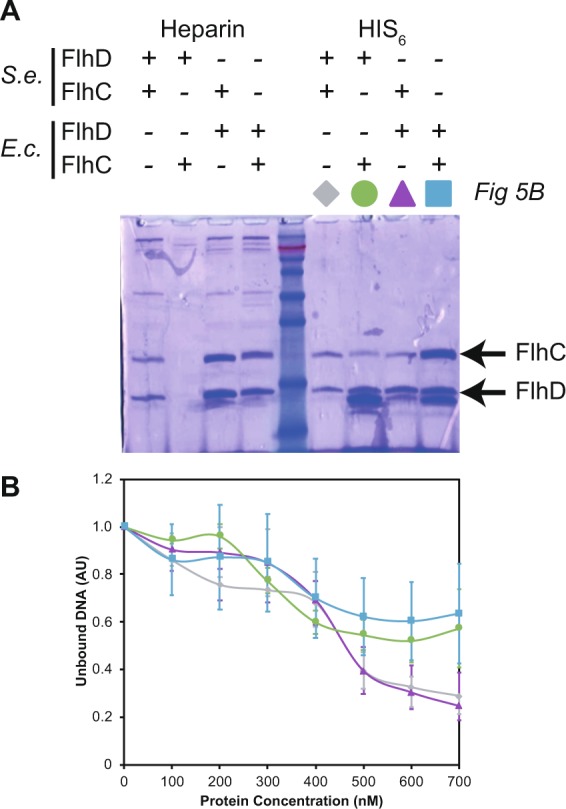
The FlhD_ST_FlhC_EC_ complex is an active but unstable complex. (**A**) Protein gel showing purified complexes with either HIS_6_ or Heparin based purification protocols. The nature of the FlhDC complex allows isolation of both proteins in these assays. Arrows indicate the FlhC and FlhD bands. The image shown is the complete gel down to the leading edge of the loading buffer. The unprocessed raw image is shown in Figure S4. (**B**) Quantification of the unbound DNA during EMSA to define the binding ability of the complex combinations compared to *S. enterica* FlhD_4_C_2_. The protein complexes used in these assays were isolated via the HIS_6_ protocol as indicated in A by the corresponding coloured symbols that the act as the key for (B). All colours reflect the same complex associated with data shown in Figs 2, 4 and 6 for continuity, for example FlhDC_SE_ is gray. Error bars show the standard error of the mean. See text for values of the calculated slopes using the excel built-in function SLOPE to highlight the impact of FlhC_EC_ in each isolated complex.

The results above demonstrate that *flhC*_EC_ is not functionally identical to *flhC*_ST_. One possibility is that that FlhC_EC_ is impaired in FlhD_4_C_2_ for DNA-binding. Alternatively, the stability of the FlhD_4_C_2_ complex is reduced in the *flhC*_*EC*_ strain, leading to reduced FlhD_4_C_2_ activity. To test these hypotheses, we purified all combinations of the FlhD_4_C_2_ complex using affinity (Ni+ and heparin) chromatography (Fig. 5A). In each complex, FlhD was tagged with a carboxy-terminal hexa-histidine to facilitate affinity purification. Such expression constructs have previously been used successfully to purify the FlhD_4_C_2_ complex^17,18^. Using either Ni+ affinity or heparin purification, we observed complete complex retrieval for three combinations (Fig. 5A). FlhC recovery was less efficient in the FlhD_SE_/FlhC_EC_ complex. In contrast, no FlhD_SE_/FlhC_EC_ complex was recovered via Heparin purification, used to mimic DNA during protein purification of DNA-binding proteins (Fig. 5A). This suggests that the FlhD_SE_/FlhC_EC_ complex is less stable, resulting on a lower yield of complex retrieval.

We next used the EMSA assays to test all four protein complexes for their ability to bind the *S. enterica* P_*flgAB*_ promoter region. Quantification of the DNA shifts showed that complexes containing the orthologous FlhC_EC_ reduced the P_*flgAB*_ promoter binding profile, compared to FlhC_SE_ complexes (Fig. 5B). This difference is exemplified when calculating the SLOPE (an excel function) of each data set. For FlhDC_SE_ and FlhD_EC_FlhC_SE_ the slopes were −906 and −784 respectively. In comparison FlhDC_EC_ and FlhD_SE_FlhC_EC_ were much shallower at −1570 and −1116 respectively. This is consistent with FlhC being the DNA binding subunit of the complex and the variation in FlhD_4_C_2_ activated promoter-binding sites between *S. enterica* and *E. coli*^19^. Therefore, these results suggest that FlhC is a key determinant of DNA binding ability. Furthermore, the reduction in FlhC_EC_ motility and flagellar gene expression in *S. enterica* is a result of the FlhD_SE_/FlhC_EC_ complex being unstable, ultimately reducing the cellular concentration of the FlhD_4_C_2_ complex.

### FlhD_4_C_2EC_ responds to proteolytic regulation

*S. enterica* and *E. coli* both regulate the FlhD_4_C_2_ complex through ClpXP-mediated proteolytic degradation. Proteolytic degradation of FlhD_4_C_2_ plays a fundamental role in facilitating rapid responses to environmental changes that require motility^20,21^. The FlhD_4_C_2_ complex has a very short half-life of approximately 2–3 minutes^22^. Proteolytic degradation of FlhD and FlhC is regulated in *E. coli* and *S. enterica* by RflP (previously known as YdiV)^23^. However, *rflP* is not expressed under standard laboratory conditions in model *E. coli* strains, suggesting that ClpXP activity is modulated in a species-specific manner^7^.

Previous work has shown that RflP delivers FlhD_4_C_2_ complexes to ClpXP for degradation^24^. We have assessed the impact on motility for ∆*clpP* and ∆*rflP* mutations (Fig. 3). The ∆*clpP* and ∆*rflP* mutants exhibited improved motility and flagellar gene expression, including the FlhD_SE_/FlhC_EC_ strain (Fig. 3A and B). These results suggest that proteolytic degradation mechanism of FlhD and FlhC, and its regulation, is common to *E. coli* and *S. enterica*.

**Figure 6:**
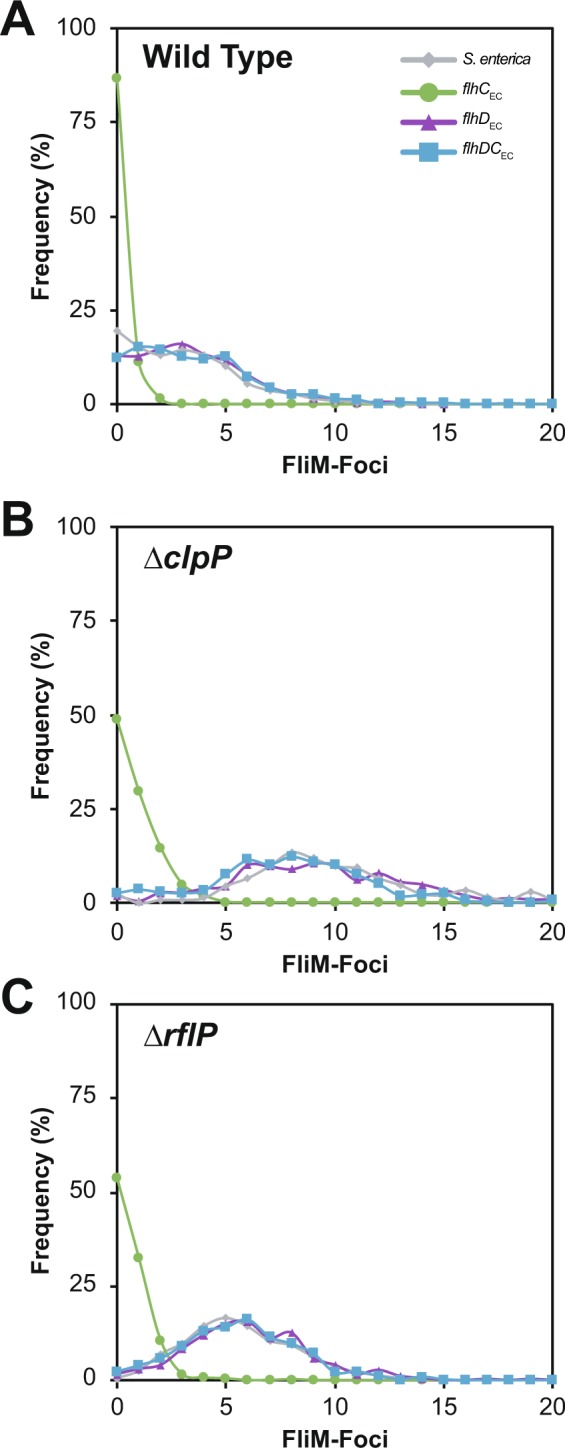
Impact of protein stability regulators of FlhD_4_C_2_ on flagellar numbers as defined by FliM-foci. Quantification of FliM-foci was performed using the semi-automatic protocols defined with in Microbetracker. (**A**) Wild Type foci distribution; (**B**) ∆*clpP*; (**C**) ∆*rflP*. All line and symbol colours reflect the same complex associated with data shown in Figs 2, 4 and 5 for continuity, for example *S. enterica* (FlhDC_SE_) is gray.

To complement the motility assays, we investigated how ∆*clpP* and ∆*rflP* mutations impact the number of FliM-foci in cell. Both ∆*clpP* and ∆*rflP* mutants showed an increased number of FliM-foci compared to the wild type (Fig. 6A–C). For *flhC*_EC_ strain, FliM-foci were observed in 13% of the population where individual cells exhibited just one or two foci. However, the ∆*clpP* or ∆*rflP* mutants increased the flagellated population of the *flhC*_EC_ strains to 51 and 46% respectively, albeit with the majority still possessing only a single FliM focus (Fig. 6 B and C).

### FliT and FliZ regulation of FlhD_4_C_2_ complexes

FlhD_4_C_2_ activity has an additional level of regulation in *S. enterica* via the flagellar-specific regulators FliT and FliZ. FliT functions as an export chaperone for the filament cap protein, FliD, and is a regulator of FlhD_4_C_2_ activity^17,25^. FliT disrupts the FlhD_4_C_2_ complex but is unable to disrupt a FlhD_4_C_2_:DNA complex. Therefore, FliT modulates availability of FlhD_4_C_2_ complexes for promoter binding^17^. In contrast, FliZ is a negative regulator of *rflP* expression^26,27^ and modulates the activity of HilD^28,29^ and thus increases the number of FlhD_4_C_2_ complexes in *S. enterica*.

In motility assays of ∆*fliT* mutants, we observed a difference between the *flhDC* strains. Motility is increased in a ∆*fliT* mutant background in *S. enterica*^30^ (and Fig. 3A). However, when *flhDC*_EC_ and *flhD*_EC_ replaced the native genes, a reduced swarm size was observed (Fig. 3A). Furthermore, quantification of P_*fliC*_ activity agreed with the motility profile for ∆*fliT* mutants, where *flhDC*_EC_ and *flhD*_EC_ containing strains had reduced promoter activity compared to wild type (Fig. 3B). This suggests that the FlhD_4_C_2_ complexes are being perceived differently by FliT in *S. enterica*. The results for ∆*clpP* and ∆*rflP* mutants suggest that this is not due to protein stability, as all complex combinations reacted in a comparable fashion (Figs 3 and 6).

In contrast, the loss of *fliZ* resulted in a consistent reduction in motility, except for the *flhC*_EC_ strain. However, as the *flhC*_EC_ strain was already impaired in motility, it is possible that the resolution of the motility assay was unable to identify differences in the ∆*fliZ* mutant. Flagellar gene expression activity did, however, suggest a 2-fold drop in P_*fliC*_ expression in the *flhC*_EC_ ∆*fliZ* strain as compared to the otherwise wild-type (Fig. 3B).

**Figure 7:**
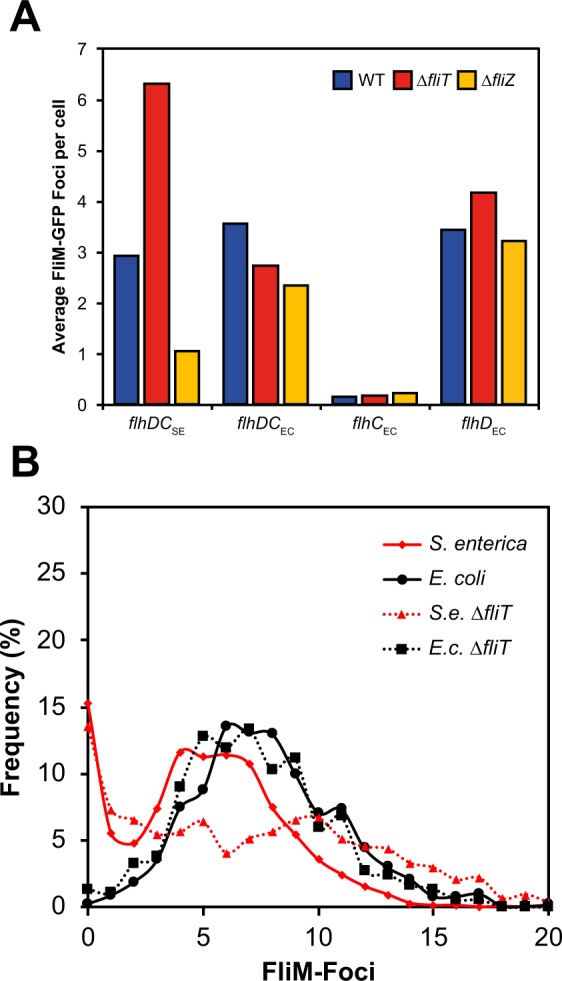
FliT and FliZ regulation reflects when FlhC_EC_ or FlhD_EC_ are present. (**A**) FliM-Foci quantification is consistent with the observed motility phenotype of ∆*fliT* mutants. For ∆*fliZ* FliM-foci numbers discriminate between the source of FlhD, FlhD_SE_ exhibits a consistnet drop in foci while FlhD_EC_ containing strains show comparable foci averages. (**B)** Testing the hypothesis that ∆*fliT* mutants respond differently in *E. coli* compared to *S. enterica*. Note: this experiment in (**B**) uses the species *E. coli* and *S. enterica* not engineered replacements.

Analysis of FliM-foci distribution in ∆*fliT* mutant reinforced the observed discrimination of *flhDC*_EC_ and *flhD*_EC_ gene replacements. Calculating the average foci per cell, *S. enterica* ∆*fliT* mutants showed an increased average number of foci per cell from 2.9 to 6.3, while the *flhD*_EC_ (*fliT*^+^: 3.4 versus ∆*fliT*: 4.2) and *flhDC*_EC_ replacements (*fliT*^+^: 3.6 versus ∆*fliT*: 2.7) exhibited no significant changes (Fig. 7A). Interestingly, in a ∆*fliZ* mutant background, the FliM-foci analysis was able to differentiate *flhDC*_EC_ and *flhD*_EC_ from the native *S. enterica flhDC* strain. Both replacements exhibited an increase in the average foci compared to *S. enterica* ∆*fliZ* (Fig. 7A).

These data suggest that there is a fundamental difference in how the FlhD_4_C_2_ complexes in *E. coli* and *S. enterica* respond to, at least, FliT regulation. There are two explanations for this: a) the *E. coli* combinations are being regulated via an unidentified mechanism in *S. enterica* or b) that they are insensitive to FliT regulation. Both arguments predict that in the species *E. coli*, FlhD_4_C_2_ may respond differently to FliT regulation. Comparing the species, not gene replacement strains, *S. enterica* and *E. coli* does indeed identify a difference in the response to a ∆*fliT* mutant. While a ∆*fliT* mutant in *S. enterica* leads to a consistent increase in FliM-foci, no significant difference is noted for an *E. coli* ∆*fliT* mutant compared to *E. coli* wild type (Fig. 7B). This suggests that the regulatory impact of FliT is very different in these two flagellar systems and the role FliT plays in *S. enterica* is potentially adaptive and species specific.

## Discussion

Two model flagellar systems that form the foundation of the flagellar field are those from the enteric species *E. coli* and *S. enterica*. These two systems have led to key discoveries in relation to many aspects of flagellar structure, type 3 secretion, flagellar cell biology and the regulation of flagellar assembly. Textbook explanations suggest that most flagellar systems are being activated, regulated and built according to the models for *E. coli* and *S. enterica*. Modifications of transcriptional regulatory circuits contribute to the phenotypic diversity we see in closely related gene sets and we are only now able to investigate this in depth due to the tools available. Here we have taken a simple step and asked how do orthologous FlhD_4_C_2_ complexes function in the closely related species *E. coli* and *S. enterica?*

At the onset of our work it was known that FlhD_4_C_2_ from *E. coli* could sustain motility in *S. enterica*^11^. Our work was focussed on understanding and defining the species-specific differences in the regulon of two orthologous genes. Here we took advantage of the well-defined flagellar assembly tools to measure outputs such as, motility, flagellar assembly per cell and flagellar gene expression. Bioinformatic analysis identifies only an 8 and 6% identity difference between FlhD and FlhC in *E. coli* and *S. enterica* respectively, suggesting that these proteins function in an analogous fashion. It is well established that related taxa usually rely on orthologous regulators to coordinate response to a given signal^10^.

The fine detail of the differences in the FlhD_4_C_2_ complexes only became apparent when we began to focus on their effect on flagellar gene expression and flagellar assembly. Biochemical analysis of isolated complexes showed that FlhC_EC_ had weaker DNA binding ability to the P_*flgAB*_ promoter region from *S. enterica*, consistent with previous investigations into FlhD_4_C_2_ DNA binding activity^19^. The isolation of FlhD_4_C_2_ complexes from our strains suggested that a key aspect of the phenotypes we observed, was the stability of the complexes formed.

With respect to *flhDC* transcription we show a discrepancy in flagellar numbers defined by FliM-foci when using P_*tetA*_/P_*tetR*_::*flhDC* expression. This was somewhat surprising as all constructs exhibited good swarming ability on motility agar plates (Figure S3). Original studies on the regulation of P_*tetA*_/P_*tetR*_ from Tn10 have shown that these two promoters have differing activities but both respond to TetR regulation. We show that even though maximal activity of P_*flgA*_ and P_*fliC*_ can reach 40–50% of P_*tetA*_::*flhDC* expression for P_*tetR*_ strains, this results in an average of 2 flagella per cell. This suggests that even though the majority of the literature states that *E. coli* and *S. enterica* produce between 4 and 8 flagella per cell, only 1 or 2 per cell is needed for an optimal output of the system with respect to motility agar assays. This conclusion correlates with the observation that swimming speed does not depend on flagella numbers in *E. coli*^31^.

It has been shown that FliT interacts with FlhC and that in *S. enterica* the output of this circuit is to destabilize FlhD_4_C_2_ complexes that are not bound to DNA. Our data suggests that this level of regulation does not impact *E. coli* FlhC. The nature of the adaptability needed by the favourable conditions to drive motility in *E. coli* may have led to the FliT regulatory input becoming less critical. Indeed, the specific amino acid substitutions between FlhC_EC_ and FlhC_ST_ merits further investigation, outside the focus of this study, to determine whether this can be defined by a single substitution or requires the combination of the changes observed between these two proteins (Figure S1). Similarly, the impact of FliZ regulation becomes apparent for FlhD_EC_ containing complexes when we assess flagellar numbers. FliZ regulates the transcription of *rflP* in *S. enterica*^27^. It is plausible that the impact in changing *rflP* regulation is the source of this differentiation, especially as RflP is proposed to interact with FlhD_SE_. Furthermore, we know that *rflP* is not expressed in model *E. coli* strains, strengthening the argument that FlhD_EC_ has adapted to the absence of RflP or vice versa FlhD_SE_ to RflP. However, regulation of flagellar gene expression in *S. enterica* via FliZ must take in to consideration other regulators such as HilD and its impact on *flhDC* gene expression^9,28,29^.

Importantly our analysis shows that even though these two systems are genetically similar, investigation of FlhD_4_C_2_ activity identifies subtle but key differences into how the FlhD_4_C_2_ complex is modulated in two closely related species. We argue that this is a valid example of the caution needed in the age of synthetic biology to exploit heterologous systems in alternative species or chassis’. Our data shows that even systems showing significant synteny may not behave in exactly the same manner and due diligence is required in making assumptions based on heterologous expression.

## Materials and Methods

### Bacterial Strains and Growth conditions

*S. enterica* and *E. coli* strains used in this study have been previously described elsewhere^12,15,17,30^. This study used *S. enterica* serovar Typhimurium strain LT2 as the chassis for all experiments. *E. coli* genetic material was derived from MG1655. All strains were grown at either 30 °C or 37 °C in Luria Bertani Broth (LB) either on 1.5% agar plates or shaken in liquid cultures at 160 rpm^17^. Antibiotics used in this study have been described elsewhere^32^. Motility assays used motility agar^17^ incubated at 37 °C for 6 to 8 hours. Motility swarms were quantified using images captured on a standard gel doc system with a ruler in the field of view and quantified using ImageJ to measure the vertical and horizontal diameter using the average as the swarm size. All motility assays were performed in triplicate using single batches of motility agar.

### Genetic Manipulations

For the replacement of *flhDC* coding sequences the modified lambda red recombination system described by Blank *et al*. (2011) was used^33^. Deletion of *clpP, rflP, fliT* and *fliZ* was performed using the pKD system described by Datsenko and Wanner (2000)^34^. P_*tetA*_/P_*tetR*_ replacements of the P_*flhDC*_ region was also performed using Datsenko and Wanner (2000) with the template being Tn10*d*Tc^35^. For Blank *et al*. (2011) replacement experiments we used autoclaved chlortetracycline instead of anhydrotetracycline as described for the preparation of Tetracycline sensitive plates^36^. All other gene replacements were performed as previously described^17^. All primers used for these genetic manipulations are available on request.

### Quantification of flagellar gene expression

Flagellar gene expression assays were performed using the plasmids pRG39::cat (P_*fliC*_) and pRG52::cat (P_*flgA*_)^15^. Both plasmids were transformed into strains using electroporation. Gene expression was quantified as described previously and analysis was based on a minimum of n = 3 repeats for each strain tested^15^.

### Quantification of FliM-GFP foci

FliM-GFP foci were quantified using Microbetracker on images captured using a Nikon Ti inverted microscope using filters and exposure times described previously^14^. Strains were grown to an OD600 of 0.5 to 0.6 and cells immobilised using a 1% agarose pad containing 10% LB^14,17^. For each strain a minimum of 5 fields of view were captured from 3 independent repeats. This allowed analysis of approximately 400–1000 cells per strain. For the comparison of FliM foci in *E. coli* ∆*fliT* to *S. enterica* ∆*fliT* shown in Fig. 7B the chemostat growth system described by Sim *et al*. (2017) was used. For this experiment the growth rate of both strains was similar to batch culture in LB at 37 °C where the media used was a Minimal E base salts, a minimal media previously described^14,17^, supplemented with 0.1% Yeast extract and 0.2% glucose.

### Purification of FlhD_4_C_2_ complexes

Purification of proteins complexes was based on previously described methods^17^. Wild type FlhD_4_C_2SE_ was purified using pPA158. The other 3 complexes were purified from plasmids generated using the New England Biolabs NEBuilder DNA Assembly kit on the backbone of pPA158. The *E. coli* strain BL21 was used for all protein induction experiments prior to protein purification using either a pre-equilibrated 5 ml His-trap column or a 5 ml heparin column (GE Healthcare). Proteins were visualised using Tricine-based SDS polyacrylamide gel electrophoresis and standard commassie blue staining^17^.

### Electrophoretic mobility shift assay (EMSA)

All EMSA assays were performed using Ni++ (his-trap) purified proteins as this allowed analysis of all four complexes (Fig. 5A). Buffer exchange from elution buffer to a 100 mM Tris-HCl, 300 mM NaCl 1 mM DTT (pH 7.9) buffer was performed through 10 cycles of protein concentration in VivaSpin columns with 20 ml buffer reduced to 5 ml per round of centrifugation at 4500 rpm. A protein concentration range of 100 to 700 nM was used with 80 ng/ml of a PCR product containing P_*flgAB*_ from *S. enterica*. After incubation bound and unbound DNA were resolved using 5% acrylamide gels made with 1x TBE buffer. Quantification of gel images was performed using ImageJ.

## Electronic supplementary material

Supplementary Information
