# Supplementary material for: Driving the expression of the Salmonella enterica sv Typhimurium flagellum using flhDC from Escherichia coli results in key regulatory and cellular differences
Source: Sci Rep. 2018 Nov 12;8:16705. doi: 10.1038/s41598-018-35005-2 (PMC6232118; doi:10.1038/s41598-018-35005-2)
Supplement: Supplementary file 1 — Supplementary Information [file 41598_2018_35005_MOESM1_ESM.pdf]

Driving the expression of the *Salmonella enterica* sv Typhimurium flagellum using *flhDC* from *Escherichia coli* results in key regulatory and cellular differences.

Ayman Albanna, Martin Sim, Paul A. Hoskisson, Colin Gillespie, Chris V. Rao and Phillip D. Aldridge*

# SUPPLEMENTARY MATERIAL

*Corresponding author. Centre for Bacterial Cell Biology, Baddiley Clark Building, Newcastle University, Richardson Road, Newcastle upon Tyne, United Kingdom, NE2 4AX. UK. Phone: +44-191-2083218. Email: phillip.aldridge@ncl.ac.uk.

**Figure S1**

## FlhD

```
S. enterica   1 MGTMHTSELLKHIYDINLSYLLLAQRLIVQDKASAMFRLGINEEMANTLGALTLPQMVKL
E. coli       1 ---MHTSELLKHIYDINLSYLLLAQRLIVQDKASAMFRLGINEEMATTLAALTLPQMVKL
consensus     1 ...******************************************* **.**********

S. enterica  61 AETNQLVCHFRFDDHQTITRLTQDSRVDDLQQIHTGIMLSTRLLNEVD---DTARKKRA
E. coli      58 AETNQLVCHFRFDSHQTITQLTQDSRVDDLQQIHTGIMLSTRLLNDVNQPEEALRKKRA
consensus    61 ************* ***** *************************.* ....  *****
```

## FlhC

```
S. enterica   1 MSEKSIVQEARDIQLAMELINLGARLQMLESETQLSRGRLIRLYKELRGSPPPKGMLPFS
E. coli       1 MSEKSIVQEARDIQLAMELITLGARLQMLESETQLSRGRLIKLYKELRGSPPPKGMLPFS
consensus     1 ******************** ********************.******************

S. enterica  61 TDWFMTWEQNIHASMFCNAWQFLLKTGLCSGVDAVIKAYRLYLEQCPQPPEGPLLALTRA
E. coli      61 TDWFMTWEQNVHASMFCNAWQFLLKTGLCNGVDAVIKAYRLYLEQCPQAEEGPLLALTRA
consensus    61 **********.****************** ******************  **********

S. enterica 121 WTLVRFVESGLLELSSCNCCGGNFITHAHQPVGSFACSLCQPPSRAVKRRKLSRDAADII
E. coli     121 WTLVRFVESGLLQLSSCNCCGGNFITHAHQPVGSFACSLCQPPSRAVKRRKLSQNPADII
consensus   121 ************ ****************************************   ****

S. enterica 181 PQLLDEQIEQAV
E. coli     181 PQLLDEQRVQAV
consensus   181 *******  ***
```

Protein alignment of FlhD and FlhC from *S. enterica* and *E. coli*. Both proteins exhibit a high level of similarity. For both proteins the greatest level of diversity occurs towards the C-termini of each protein. How these amino acid changes impact the activity of the $FlhD_4C_2$ complex is still unknown. However, the data presented in the current study can act as a foundation to investigate their impact.

**Figure S2**

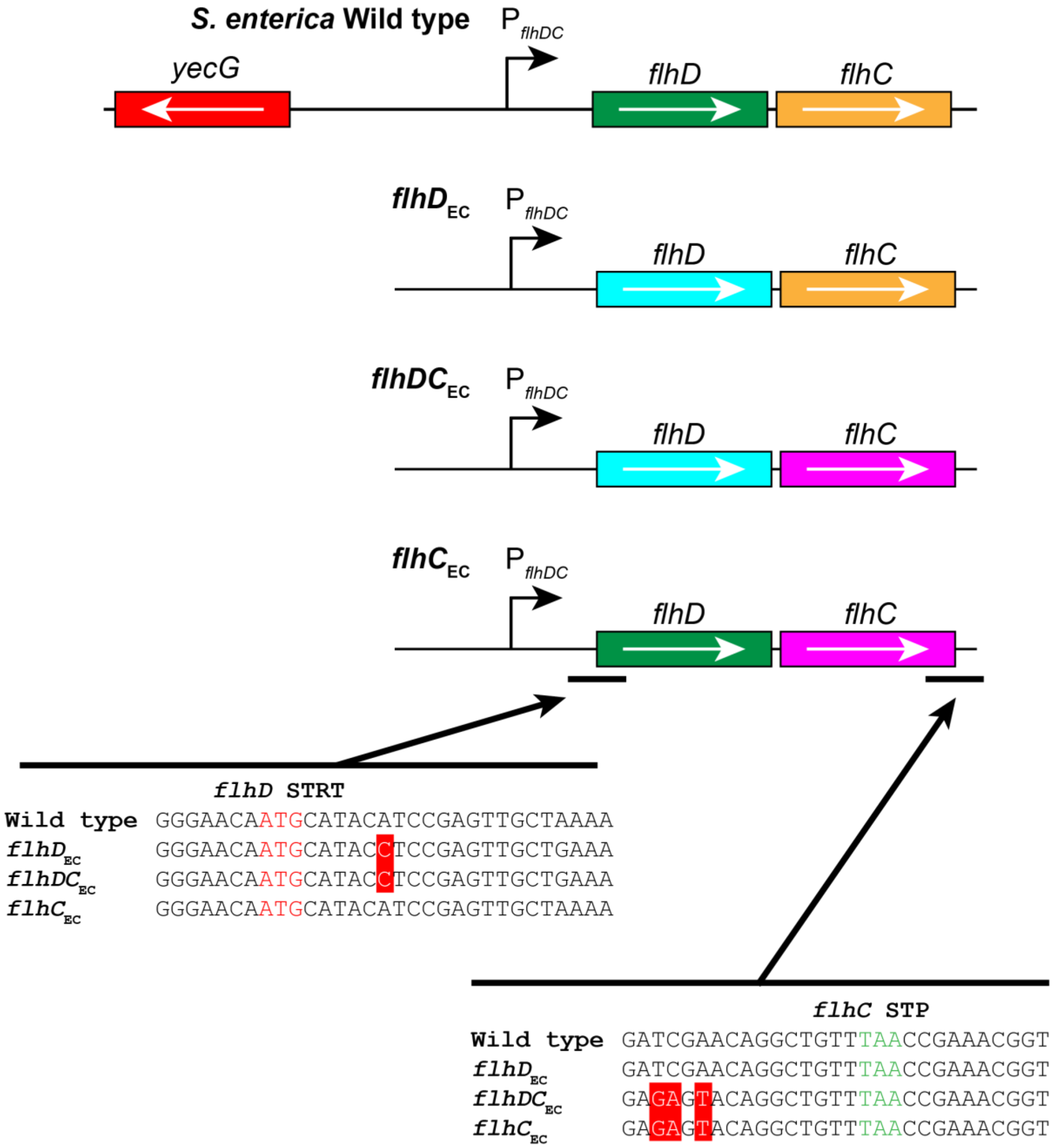


Schematic diagram detailing the 3 replacements made during this study. The *flhD* ATG start (STRT) codon boundary and the *flhC* STOP (STP) codon boundaries are shown as examples of the replacement design. Known nucleotide changes for *E. coli* in these short regions are highlighted as red boxes with white text.

**Figure S3**

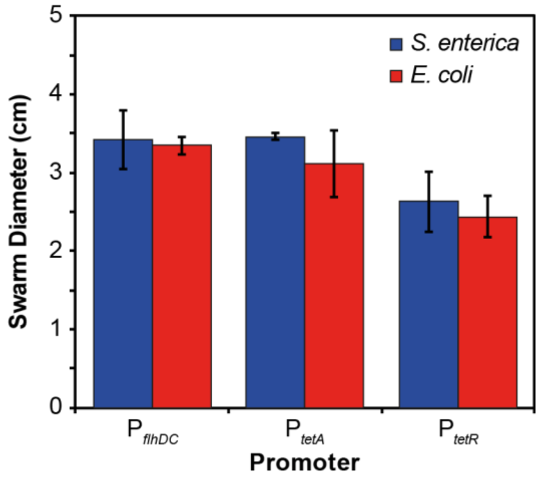


Motility of *flhDC*$_{ST}$ and *flhDC*$_{EC}$ driven by $P_{flhDCSE}$ compared to $P_{tetA}$ or $P_{tetR}$ expression. Quantification of swarms produced in motility agar after 6 to 8 hours incubation. Error bars indicate calculated standard deviations.

**Figure S4**

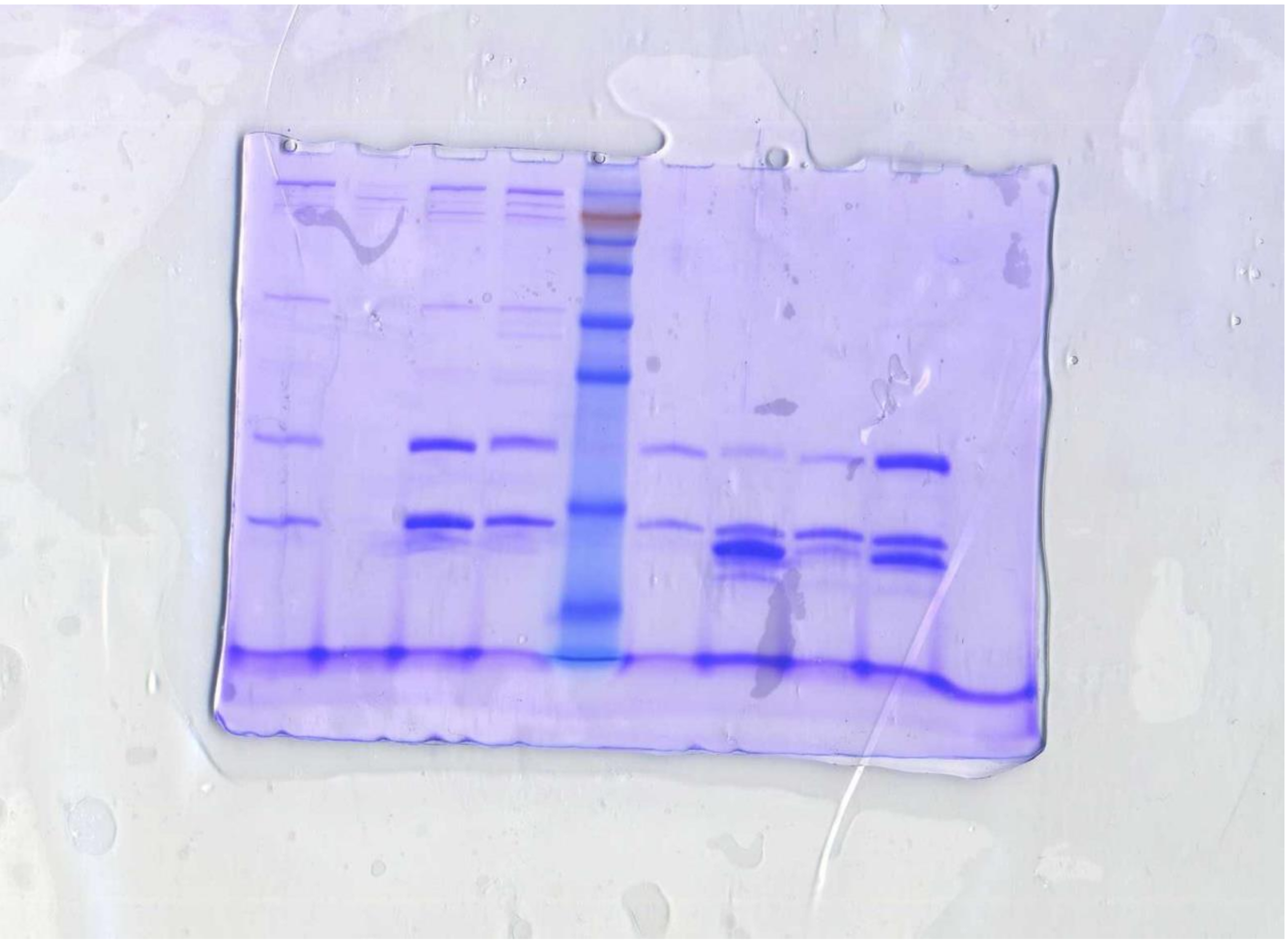

Unprocessed image of Gel shown in **Figure 5A**
